# Inhibition of miR301 enhances Akt-mediated cell proliferation by accumulation of PTEN in nucleus and its effects on cell-cycle regulatory proteins

**DOI:** 10.18632/oncotarget.7996

**Published:** 2016-03-08

**Authors:** Mayur V. Jain, Ahmad Shareef, Wirginia Likus, Artur Cieślar-Pobuda, Saeid Ghavami, Marek J. Łos

**Affiliations:** ^1^ Department of Clinical & Experimental Medicine, Division of Cell Biology Integrative Regenerative Medicine Center (IGEN), Linköping University, Linköping, Sweden; ^2^ Department of Human Anatomy, School of Medicine in Katowice, Medical University of Silesia, Katowice, Poland; ^3^ Institute of Automatic Control, Silesian University of Technology, Gliwice, Poland; ^4^ Department of Human Anatomy and Cell Science, College of Medicine, Faculty of Health Sciences, University of Manitoba, Winnipeg, Canada; ^5^ Department of Pathology, Pomeranian Medical University, Szczecin, Poland; ^6^ LinkoCare Life Sciences AB, Linköping, Sweden

**Keywords:** miR301, PTEN, PI3K, AKT, mTOR

## Abstract

Micro-RNAs (miRs) represent an innovative class of genes that act as regulators of gene expression. Recently, the aberrant expression of several miRs has been associated with different types of cancers. In this study, we show that miR301 inhibition influences PI3K-Akt pathway activity. Akt overexpression in MCF7 and MDAMB468 cells caused downregulation of miR301 expression. This effect was confirmed by co-transfection of miR301-modulators in the presence of Akt. Cells overexpressing miR301-inhibitor and Akt, exhibited increased migration and proliferation. Experimental results also confirmed PI3K, PTEN and FoxF2 as regulatory targets for miR301. Furthermore, Akt expression in conjunction with miR301-inhibitor increased nuclear accumulation of PTEN, thus preventing it from downregulating the PI3K-signalling. In summary, our data emphasize the importance of miR301 inhibition on PI3K-Akt pathway-mediated cellular functions. Hence, it opens new avenues for the development of new anti-cancer agents preferentially targeting PI3K-Akt pathway.

## INTRODUCTION

Micro-RNAs (miR) are small non-coding RNAs that act as key regulators of cellular and supracellular (for example immune response) processes under normal and diseased conditions. The importance of miRs in cancer has been underlined by controlling the expression of target mRNAs to facilitate carcinogenesis [1]. Cancer promoter or suppressor function of miRs depends on their direct target genes. As an example, it has been showed that miR155 [2], miR21 [3], miR300 [4] promote breast cancer, while miR7 [5], miR16 [6], miR30a [7] are involved in its suppression. Recently, miR301 has attracted much attention due its significant contribution to different biological process including differentiation, proliferation, survival, apoptosis in pancreatic- [8], hepatocellular- [9], lung-, colorectal- [10], breast cancer [11] and in sickle cell disease [12].

Several miRs target signaling molecules like TGF-β, Wnt, EGF and Akt, and they serve as nodes for signaling pathways that regulate the central cellular processes such as cell survival, proliferation, transcription, translation, cell cycle, differentiation and metabolism [13]. PI3K-Akt signaling pathway is hyper-activated in majority of cancers because of the frequent mutation of tumor suppressor gene, PTEN [14]. PTEN is a central negative regulator of PI3K-Akt signal transduction by dephosphorylating PI(3,4,5)P3 and inhibiting downstream signals [14]. Although PTEN has well-delineated function in the cytoplasm, it has also been found in the nucleus, under certain conditions. Nuclear function of PTEN may be in opposition to its cytoplasmic function [15].

PI3K is a heterodimeric complex containing a separate regulatory- (P85) and catalytic subunit (P110) which phophorylates the inositol ring of PI(4,5)P2 at third positions to create PI(3,4,5)P3 and that serves as an anchorage site for signaling molecules [16]. This function of PI3-Kinase is essential for the recruitment of particular proteins containing pleckstrin-homology (PH) domain or FYVE domain to the cellular membrane [17]. Akt/PKB, a primary downstream signal-integrator and transducer carries the PH domain, and is recruited to the plasma membrane [18, 19]. Membrane recruitment and binding to PI(3,4,5)P3 causes conformational changes in Akt, resulting in exposure of its phoshorylation sites T308 and S473 which are then phosphorylated by phosphoinositide dependent kinase (PDK) and mTOR/Rictor [18–20]. The PI3K/Akt signaling cascade affects (indirectly) the regulation of over 800 downstream signaling molecules that play a role in cell survival, growth, migration, proliferation and cell death [18, 19].

Recently, we have reported that Akt and its nuclear localization play an important role in maintenance and proliferation of cancer stem-like cells through the counter-regulation of p21^Waf1/Cip1^ and p27^kip1^ [21]. Nuclear and cytoplasmic Akt pools play partially opposing roles. Nuclear Akt directly inhibits the activity of CDK2-inhibitor p27^kip1^, whereas it indirectly inhibits the activity of anti-apoptotic molecules such as Bcl2 (by increasing the activity of CDK2). Akt also may inhibit forkhead transcription factor FKHR and promote the activation of anti-apoptotic molecules [22]. The function of miR301 in regulation of PI3K-Akt signaling pathway is poorly understood; therefore in the present study we have investigated the effect of miR301 on Akt mediated biological functions in breast cancer cells, when miR301 expression was decreased upon Akt-overexpression.

## RESULTS

### Akt overexpression downregulates miR301 level in breast cancer cells

**Figure 1:**
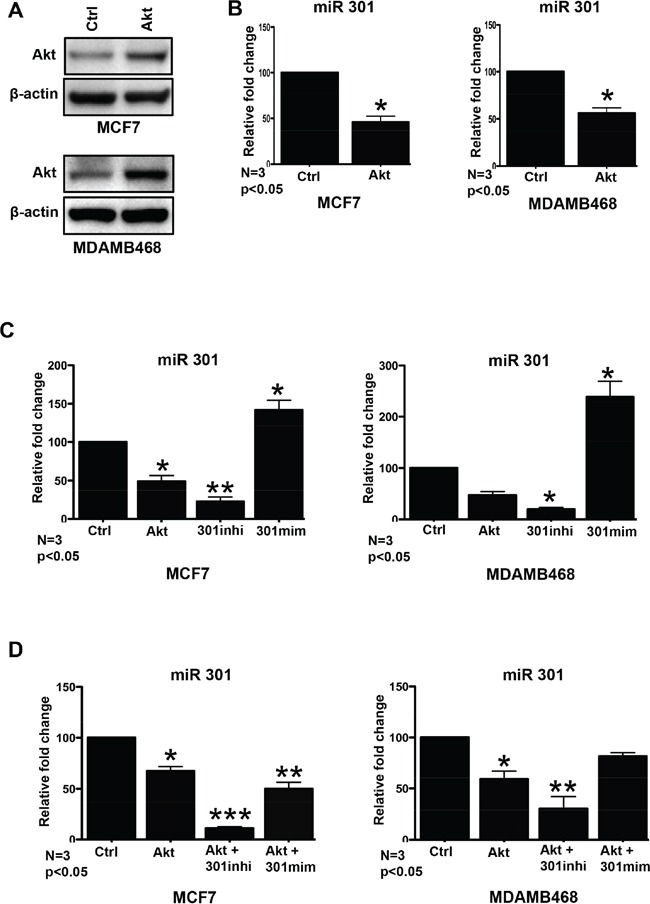
Effect of miR301 on PI3K-Akt pathway **A.** In order to study changes in miR levels upon Akt upregulation cells were transiently transfected with Akt. Western blot confirms overexpression of Akt as compared to empty plasmid (control) in MCF7 and in MDAMB468 cells. **B.** Quantitative RT-PCR shows significant downregulation of miR301 expression mediated by transient transfection with Akt construct, as compared to cells transfected with empty vector, in MCF7 and MDAMB468 cells **C.** Quantitative RT-PCR shows significant downregulation miR301-expressions by Akt overexpression, even more downregulation by miR301 inhibitor and upregulation by miR301-mimic, as compared to Ctrl (negative control) miR in breast cancer cells. **D.** Co-transfection of Akt construct with miR301-modulators and Akt alone shows downregulation of miR301 expression in comparison with the control (empty plasmid and Ctrl miR). Since the effect is observed regardless of use of miR301-inhibitor or -mimics, it is likely due to Akt-overexpression, and confirms our results from (A). Akt transfection with miR301-inhibitor showed strong downregulation of miR301 expression compared to control (* p < 0.05).

In the manuscript, we refer to Akt-1 as Akt, and miR301-3p as miR301. Exiqon miRCURY LNA^™^ Array was used to conduct microRNA profiling of breast cancer cell lines (MCF7, HEK293, SKBR3) transiently transfected with Akt. The results showed that miR301 expression was downregulated on average 3.5 fold in MCF7 and HEK293. The transient overexpression Akt was confirmed by immunoblotting (Figure 1A). We further confirmed by qPCR, that miR301 expression is significantly decreased in Akt transfected MCF7 and MDAMB468 cells (Figure 1B).

Once the miR301 downregulation by the transfection of Akt construct was established, we focused on the biological role of miR301. The miR301 specific inhibitor and mimic were utilized to knockdown and boost the miR301 expression in breast cancer cells (Figure 1C). Furthermore, we checked the effect of co-transfection of miR301 inhibitor or mimic with Akt construct and observed that miR301 mimic with Akt showed downregulation of miR301 expression similar to Akt construct alone in MCF7 (Figure 1D). A similar trend was observed in MDAMB468 cells.

### Effect of Akt mediated downregulation miR301 on breast cancer cell survival and migration

**Figure 2:**
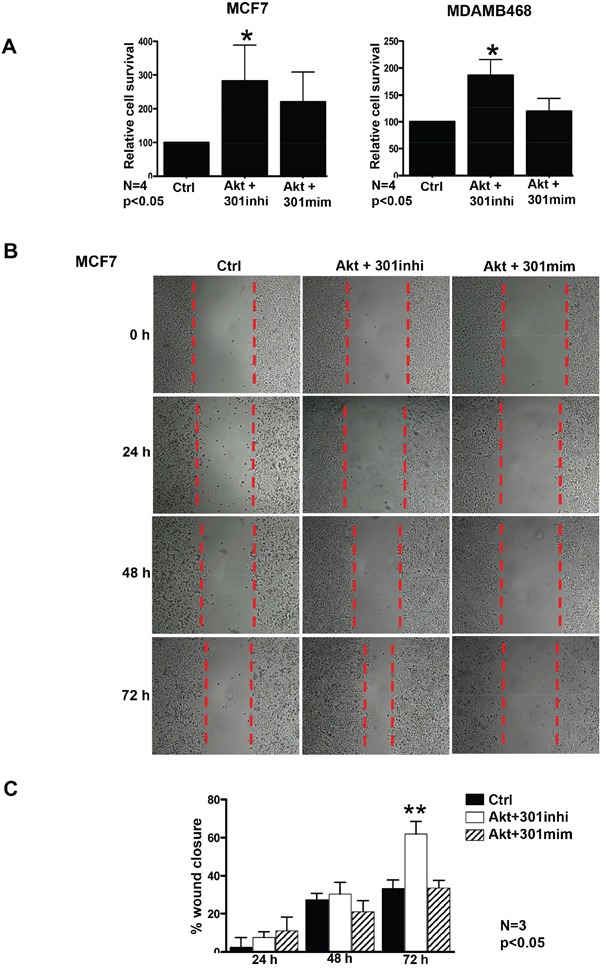
Akt mediated increase in cell survival and migration upon down-regulation of miR301 **A.** MTT-assay was performed to measure the cell survival upon downregulation of the miR301 with Akt. MCF7 and MDAMB468 breast cancer cells transiently transfected with miR301 inhibitor in the presence of Akt shows a significant increase in cell survival compared to control. **B.** Scratch assay was preformed to assess cell migration upon inhibition of miR301 in Akt-overexpressing cells. 72h post transfection, the cell migration was highely pronounced in cells co-expressing miR301 inhibitor and Akt, as compared to control, in MCF7 cells (* p < 0.05). **C.** Quantitative assessment of scratch shows significantly higher wound closure at 72h post transfection, upon miR301 inhibition in the presence of Akt compared to control in MCF7 cells (* p < 0.05).

Given the strong association between miR301 inhibition and Akt, we have investigated whether miR301 inhibition could have any effect on cell survival and migration. Previously, it has been shown that Akt-mediated upregulation of cell survival pathways plays a key role in drug resistance [18, 19, 23]. Here, we evaluated the role of miR301 in metabolic/mitochondrial activity (not necessary proliferation) by MTT assay. MCF7 and MDAMB468 cells were separately co-transfected in 96 well plates, with Akt and miR301-modulators. We observed that downregulation of miR301 expression by Akt and miR301 inhibitor resulted in significant increase in cell viability as compared to control (Figure 2A). Additionally, cell migration was accessed by the scratch assay (known also as ‘wound-healing assay’). Cell migration undeniably increased by transfection with a vector expressing Akt with miR301 inhibitor, as compared to control in MCF7 cells (Figure 2BC).

### miR301 inhibition causes increased proliferation of breast cancer cells

**Figure 3:**
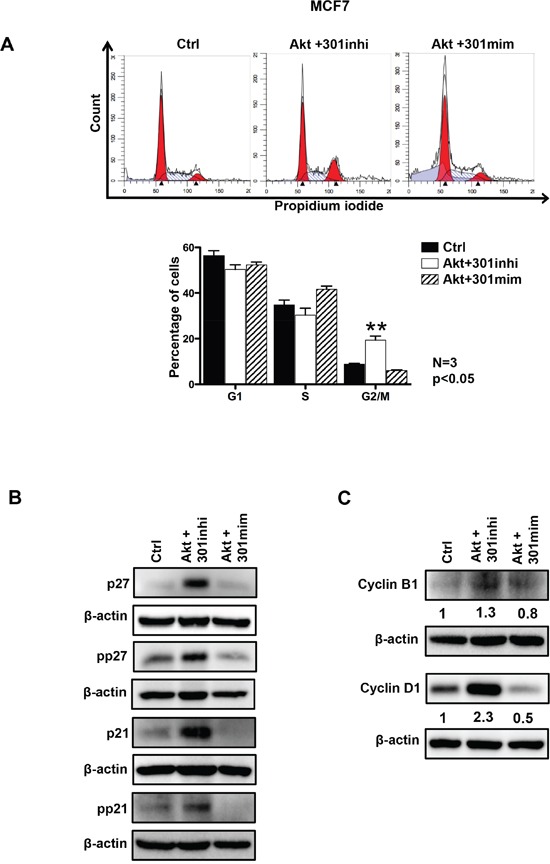
Role of miR301 in the presence of Akt on cell cycle progression **A.** Cell cycle analysis shows significant increase in the G2/M phase upon inhibition of miR301 expression in the presence of Akt compared to control and miR301-mimic+Akt in MCF7 breast cancer cells. Lower panel represents quantification of data (**A**). **B.** Western blot represents detection of p21^Waf1/Cip1^ and p27^kip1^ cell cycle inhibitory proteins. The levels of total and phophorylated p21^Waf1/Cip1^ and p27^kip1^ protein were increased upon inhibition of miR301 in the presence of Akt compared to control and miR301-mimic+Akt in MCF7 cells. **C.** Western blot analysis of Cyclin D1 and Cyclin B1 cell cycle regulatory proteins. Levels of Cyclin D1 and Cyclin B1 were increased upon the expression of miR301 inhibitor in the presence of overexpressed Akt, as compared to control, in MCF7 cells (* p < 0.05).

We assessed the impact of miR301 inhibition or overexpression, in combination with Akt overexpression, on cell cycle profile in breast cancer cells. As shown in the Figure 3A, miR301 inhibition in cells overexpressing Akt, caused decrease of cells in G1 phase and significant increase in G2 and mitosis as compared to control. Co-transfection of miR301-mimic with Akt presented opposite effect on cell cycle distribution (Figure 3A, Supplementary Figure 1A). We have recently shown [21], that Akt nuclear accumulation causes decrease in G1 phase of the cell cycle by phosphorylation of p21^Waf1/Cip1^ which leads to decrease p21^Waf1/Cip1^'s cell cycle inhibitory function. This event ultimately causes the nuclear export of p21^Waf1/Cip1^ [24]. In the current study, we checked the protein expression by Western blot, and we observed that the co-transfection of miR301 inhibitor with Akt, in breast cancer cells resulted in significant increase in total and phosphorylated p21^Waf1/Cip1^ and p27^kip1^ protein levels (Figure 3B and Supplementary Figure 1B).

To get a broader insight of the effect of miR301 on proliferation, cell cycle regulatory proteins were studied in the miR301 inhibited Akt-overexpressed cells. We observed a strong upregulation of the expression of Cyclin D1, (involved, among others, in the regulation of G1/S transition) upon co-transfection of miR301 inhibitor and Akt (Figure 3C). We further analyzed Cyclin B1 expression, which regulates the S/G2, and mitosis entrance. Cyclin B1 expression was upregulated by Akt and miR301 inhibitor further upregulated its expression in MCF7 cells (Figure 3C).

### Effect of miR301 on cell survival of breast cancer cells

**Figure 4:**
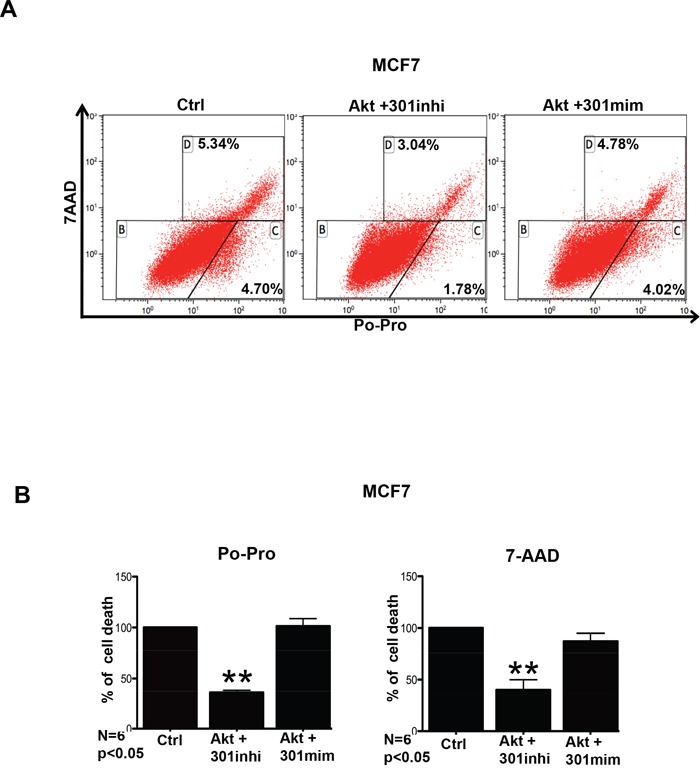
miR301 inhibition in the presence of Akt reduced cell death **A.** Cell death was assessed using Po-Pro (apoptotic) and 7-AAD (necrotic) markers. Transient transfection of miR301 inhibitor and Akt resulted in reduced cell death compared to control MCF7 cells. **B.** Quantitative representation of data from “**A**” shows significantly decreased in cell death as compared to control (* p < 0.05).

We assessed whether the cell survival functions of Akt is enhanced in our model upon inhibition of miR301. Thus, we checked how Akt combined with miR301-modulation affects cell death, using the combination of apoptotic dye Po-Pro and necrotic cell death marker 7-AAD. As shown in Figures 4A, B, cells overexpressing miR301 inhibitor together with Akt showed lower staining for Po-Pro and 7-AAD, implying an increase in cell survival in the presence of miR301 inhibitor with Akt overexpression in MCF7 cells, whereas co-expression of Akt and miR301-mimic had an opposite effect. It is worth to note that although the observed changes were statistically significant (p < 0.05) they were not very strong.

### Effect of miR301 on other known miR301-target proteins in breast cancer cells

**Figure 5:**
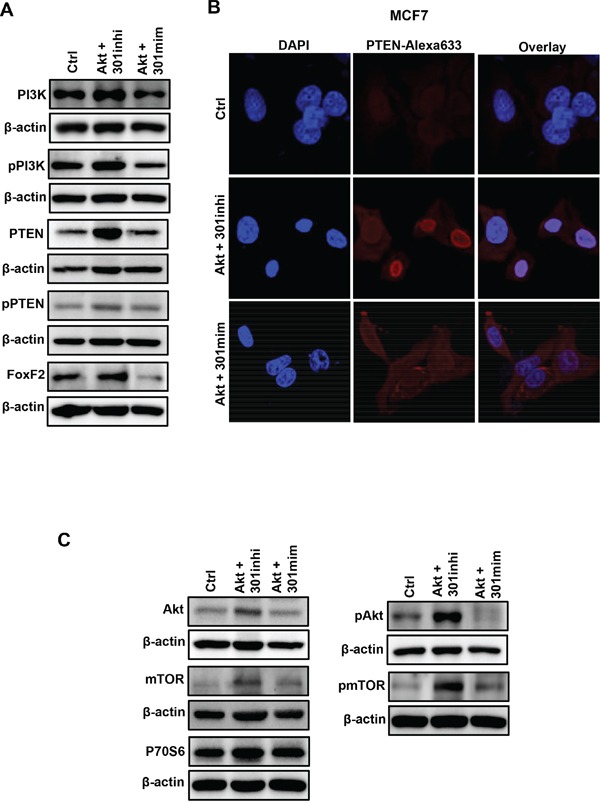
miR301 targeted gene expression in breast cancer cells **A.** Western blot analysis of miR301 targets PI3K, PTEN and FoxF2 indicates that total protein levels were significantly increased upon miR301 inhibition in Akt-overexpressing MCF7-cells, as compared to control. Phosphorylated PI3K levels were increased as compared to control and phosphorylated PTEN protein expression was not significantly changed in cells expressing miR301-modulators and Akt. **B.** Confocal-microscopy images show enhanced nuclear localization of PTEN (*red*) in MCF7-cells transiently-transfected with miR301 inhibitor and Akt. Transfection with miR301 mimic and Akt caused PTEN's cytoplasmic localization, in MCF7 cells. For better visualization of nuclei, cells were counterstained with DAPI (*blue*). **C.** Western blot analysis shows increased levels of phosphorylated Akt (pAkt), pmTOR and P70S6 in MCF7 cells transiently transfected with miR301 inhibitor and Akt.

Our data indicates that miR301 inhibition promotes diverse Akt-mediated effects including proliferation, survival and migration. Thus, we investigated the effect of miR301-modulation on other miR301-targets in our experimental system. Western blot analysis showed the increased protein expression of PTEN, PI3K and FoxF2, as compared to control upon combined miR301 inhibition with Akt overexpression (Figure 5A). Intriguingly, PI3K is the upstream regulator of Akt activity, and plays an important role in Akt activation, whereas PTEN is a negative regulator of Akt pathway. Given the observed similarity in the expression of PI3K and PTEN among miR301 inhibitor with Akt transfected cells, we further checked the protein levels of activated/phosphorylated PI3K and PTEN. We have observed a marked upregulation of phosphorylated PI3K level upon miR301 inhibition in Akt-overexpressed cells. In contrast, the level of phosphorylated PTEN was not affected upon miR301 inhibition or overexpression in the presence of Akt-overexpression (Figure 5A, and Supplementary Figure 2A). We next checked the intracellular localization PTEN upon miR301 inhibition in the presence of Akt-overexpression. miR301 inhibition in Akt-overexpressing cells increases PTEN-nuclear localization. Fraction of PTEN still remains in the cytoplasm (Figure 5B). Co-transfection of miR301-mimic and Akt lead to mostly cytoplasmic localization of PTEN (Figure 5B).

The modulation of miR301 had strong effect on PTEN-localization hence we next focused on the functional aspects of PTEN intracellular compartmentalization. Since PTEN localized in the nucleus is unable to dephosphorylate PIP3 to PIP2, it does not counteract the activation of Akt-activation [15]. This observation was further confirmed by increased protein levels and phosphorylation of Akt and its downstream target, mTOR, and to a lesser extend P70S6 (Figure 5C, and Supplementary Figure 2B).

## DISCUSSION

**Figure 6:**
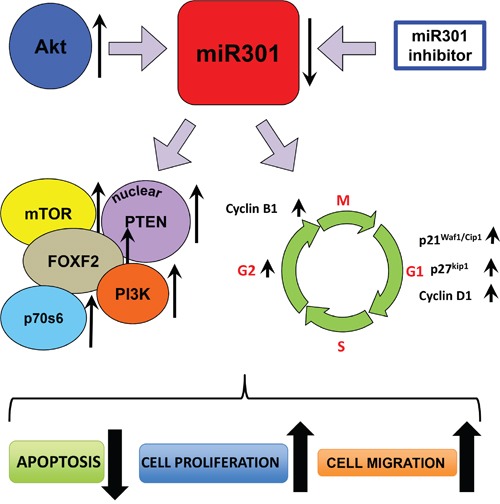
Schematic outline of the miR-301's functions in breast cancer cells In the proposed model overexpression of Akt causes down-regulation of miR301. This effect can be also reached and strengthened by applying miR301 specific inhibitor. Down-regulation of miR301 leads to the increase of expression of PI3K, FOXF2, mTOR, p70s6 and nuclear localized PTEN. At the same time several cell cycle related proteins (p21^Waf1/Cip1^, p27^kip1^, Cyclin D1 and Cyclin B1) are also overexpressed, which results in higher proliferation rate and accumulation of cells in G2 phase. This all cause increase of cell migration and prevents the induction of apoptosis.

In the last decade, deregulatory role of miRs have been widely accepted and reported in majority of cancers [25]. Evidence suggests that miR expression pattern is strongly associated with the specific characteristics of malignant cells, and could be used for distinguishing normal cells and malignant ones [26–28]. Previous studies have revealed that miR has potential to serve as either tumor promoter or suppressor and modulate diverse cellular pathways by downregulation or upregulation of various genes. In the present study, we have shown that miR301 expression is significantly decreased in breast cancer cells upon Akt transient transfection. Previous studies have shown that deregulation of the PI3K-Akt-mTOR signaling pathway contributes to numerous hallmarks of tumor progression, like tumor-angiogenesis, cell proliferation, survival and migration in various cancers [29]. These effects are usually mediated via inhibition of pro-apoptotic signaling molecules and enhancement of the cancer stem-like cells survival [21, 30]. As miR and Akt are known to have diversified cellular functions in oncogenesis, we aimed to explore the miRs role on functional aspects of Akt in human breast cancer cells. Our data shows that overexpression of Akt in breast cancer cells caused downregulation of miR301 expression. Since miR301 mostly counteracts Akt effects, miR301-downregulation upregulates PI3K-Akt pathway. The inhibition of miR301-expression is associated with an increased Akt mediated cell survival, proliferation and migration (Figure 6). These pleiotropic effects of miR301 on PI3K-Akt pathway are directly correlated with the modulation of oncogenic targets including PI3K, PTEN and FoxF2 (Figure 5A). In the current study, PI3K emerges to be the crucial target of miR301 in breast cancer cells. PI3K synthesizes the PIP3 from PIP2, which attracts to the plasma membrane and promotes activation of Akt [16]. Phosphorylated Akt affects the activity of over 800 proteins. It is also the key modulator of mTOR/P70S6 kinase cascade; key autophagy regulators [18, 19, 31, 32]. Our result shows significant increase of Akt activity, mTOR, and P70S6-phosphorylation in cells transfected with miR301-inhibitor that overexpressed Akt. These findings indicate that miR301 plays an important role in the negative modulation of PI3K-Akt pathway.

One of the prominent targets of miR301 is PTEN, a tumor suppressor gene and negative regulator of PI3K-Akt pathway. Shi W., *et al* has recently shown that there is a strong correlation between miR301 and PTEN expression in human breast cancer patients [11]. Phosphorylated PTEN counteracts PI3K-signalling by converting PIP3 back to PIP2, on plasma membrane [33]. In numerous cancer cell types large proportion of PTEN localizes into the nucleus [34]. Nuclear PTEN is unable to counteract PI3K-signalling. Intriguingly, nuclear PTEN function is not yet clearly understood, however, a recent study reported that nuclear-localized PTEN does not dephosphorylate PIP3 [15]. We show that miR301 inhibition both enhances PTEN expression and its nuclear localization. miR301 inhibition has however no effect on PTEN-phosphorylation in breast cancer cells.

Our study also sheds new light on the potential functions of FoxF2 an another target of miR301. FoxF2 is a transcription factor involved in the regulation of different cellular functions [35]. Its role in cancer is not completely understood. Previous studies have reported that there is a correlation between FoxF2 and Wnt5a expression [36]. Wnt5a's role in human cancer is controversial; it can function both as cancer negative regulator [37] and oncogenic factor [38] in a context-dependent manner. Our work shows significant increase of FoxF2 expression upon miR301 inhibition when Akt expression is upregulated. Thus, our data suggest FoxF2's role as a tumor promoter, however further studies are required to clarify this aspect.

One of the foremost functions of PI3K-Akt is the induction of cell proliferation through the phosphorylation of cell cycle inhibitory proteins p21^Waf1/Cip1^ and p27^kip1^ [39, 40]. Akt also leads to an increase in the levels of cell cycle promoters: cyclin D1 and cyclin B1 [41–43]. Since Akt may affect the status of cell cycle-regulating proteins, we have investigated whether miR301 inhibition in cells overexpressing Akt affects cell cycle progression in breast cancer cells. Indeed, the miR301 inhibition in conjunction with Akt-overexpression, shortens the G0/G1 phase and relatively increases the percentage of cells in G2. In agreement with the above, we have observed increased phosphorylation of p21^Waf1/Cip1^ and p27^kip1^ upon miR301 inhibition, leading to p21^Waf1/Cip1^ and p27^kip1^ cytoplasmic translocation and removal of its inhibitory effect on cell cycle progression. Based on these evidences, we further looked for the role of miR301 in the cell cycle. miR301 inhibition in Akt-overexpressing cells lead to an increase in Cyclin D1 and -B1 protein expression. Thus, miR301 inhibition enhances Akt-mediated promotion of proliferation.

In conclusion, our study has shown a novel Akt-PI3K pathway inhibitory role of miR301 in breast cancer cells through regulation of PI3K, PTEN and FoxF2. The resulting phenotype triggered by miR301 inhibition includes increased cell survival, migration and proliferation. The data also suggest that the miR301-analogues could serve as leads for the development of PI3K/Akt pathway modulators.

## MATERIALS AND METHODS

### Cell culture and reagents

Breast cancer cell lines: MCF7, MDAMB468, SKBR3 and HEK293 were cultured in DMEM media (PAA, Pasching, Austria) containing 10% fetal bovine serum (PAA, Pasching, Austria) and 1% penicillin-streptomycin (Gibco, USA) and incubated at 37°C with 5% CO_2_ in a humidified atmosphere.

### Antibodies

The primary antibodies used in the study: pPI3K110 obtained from Bioss Antibodies (USA), PTEN, pPTEN, P70S6, Cyclin B1, pAkt, Akt1, pmTOR and mTOR from Cell Signaling (Beverly, USA), FoxF2, PI3K110, Cyclin D1, ß-actin, p27 and p-p27 from Abcam (Cambridge, UK), and p21 and p-p21 from Santa-Cruz (USA). The secondary antibodies: Alexafluor 633 obtained from Life Technologies, anti-rabbit HRP-conjugate from Biorad (USA) and anti-mouse HRP-conjugate from GE Heath Care (Buckinghamshire, UK).

### Plasmids and transient transfection

The cells were co-transected using X-treme GENE HP DNA Transfection Reagent (Roche, Mannheim, Germany) according to manufacture's instructions. Akt1 cDNA was cloned into pLVX-Tight-Puro (Clontech) plasmid as previously reported [21] and empty plasmid pLVX-Tight-Puro was used as control. *mir*Vana 301miR inhibitor, *mir*Vana 301miR mimic as a positive control and *mir*Vana miR negative control (Ambion) were all transfected at a final concentration 30 nmol/L.

Terminology explanation: *miR301 mimic-* miR301 mimic is small, chemically modified doubled-stranded RNA molecule that mimics the actions of endogenous miR301; *miR301 inhibitor* – miR301 inhibitor is small, chemically modified single-stranded RNA molecule designed to specifically bind and inhibit endogenous miR301; *Negative control* – The negative control is a random sequence of miR molecule which has been extensively tested in human cell lines and validated not to produce identifiable effect of known miR function. This negative control transfected sample has been used as a baseline for evaluating the effect of control and experimental miR301 mimic or inhibitor expression.

### Target identification by miR profiling

MCF7, SKBR3 and HEK293 cells were transfected with Akt or empty plasmid (control). In a pilot experiment, transient transfection efficiency was assessed by transfections with pEGFP (Invitrogen) vector and subsequent measurement of green fluorescence by flow cytometry. The following efficiency was achieved: MCF7 40%, HEK293 55%, and SKBR3 25%. RNA was isolated and sent to EXIQON for microRNA profiling by miRCURY LNA^™^ Array. microRNA with greater than 2-fold expression change relative to control were considered as potential targets and selected ahead for further investigation. The investigated here miR301 was down-regulated about 3.5 fold in two (MCF7, HEK293) tested cell lines. It was the most down-regulated miR, and thus it was chosen for further investigation.

### Quantitative real time-PCR analysis of miRs

For the quantitative real time-PCR, total RNA was extracted using High Pure miRNA isolation Kit (Roche, Germany) according to the manufacturer's instructions from co-transfected breast cancer cells. Total RNA concentration was measured by NanoDrop™ spectrophotometer (Thermo Scientific, USA). Total RNA was reverse transcribed into cDNA using TaqMan® MicroRNA Reverse Transcription kit (Life Technologies Ltd, USA) using CFX96™ real-time PCR detection system (Biorad, USA). Next, cDNA was added to the reaction mix containing TaqMan® Fast Universal PCR Master Mix and TaqMan MicroRNA Assay for miR301. All experimental procedures were performed according to the manufacturer's protocols. Each reaction was performed in triplicates using a CFX96™ real-time PCR detection system (Biorad, USA). RNU6b was used as internal control to normalize the amount of cDNA between different samples. The expression of miR-301 was calculated by applying the 2^−ΔΔCt^ method, where ΔCT = (CT_miR_- CT_miR control_) and CT (i.e. threshold cycle) indicates the fractional cycle number at which the amount of amplified target reaches a fixed threshold [44].

### Cell migration assay

For scratch migration assay, miR301 inhibitor and mimic with Akt co-transfected cells were scratched (wound) using a sterile standard 200 ml tip in 6-well plate. Serial images were taken at different time points using JuLi Smart Fluorescence cell imager in the bright field. The degree of ingrowth of the cells into the “wound” (scratch) indicated the proliferative capacity of cells [23].

### Cell survival assay

Cell survival and proliferation was assessed by 3-(4,5-dimethyl-2-thiazolyl) 2,5-diphenyl-2H tetrazolium bromide (MTT) assay. Cells (5000/well) were plated 24h prior to the experiments in a 96-well plate. 48 hours after co-transfection with miR301 inhibitor, mimic, and with Akt, cells were incubated with 10 μl 5 mg/ml MTT solution (Sigma Aldrich) for 3h. Next, plates were centrifuged and supernatant was discarded, formazan crystals were dissolved in DMSO:ethanol (1:1 ratio). The readings were performed at 570 and 630 nm using a spectrophotometer as described previously [23]. Data was always collected in triplicates.

### Cell cycle analysis

The experiments were preformed as described previously [45], with minor modifications. Briefly: cells were trypsinized and fixed with ice cold 70% ethanol overnight at −20°C and washed with PBS and resuspended in 50 μg/ml RNAse for 30min at 37°C. Then samples were incubated with 100 μg/ml propidium iodide (Sigma) for 10min and measured by flow cytometry (Gallios, Beckman Coulter Inc.). Kaluza software (Beckman Coulter Inc.) was used to analyze the results.

### Po-Pro and 7AAD cell death assay

Co-transfected cells were trypsinized and washed with PBS. Cells were then resuspended in PBS and treated with Po-Pro and 7AAD dyes (Life Technologies Ltd, USA) for 30 min according to the manufacturer's instruction. The fluorescence intensities of the samples were quantified using Gallios flow cytometer (Beckman Coulter Inc.) and the data was analyzed using Kaluza software (Beckman Coulter Inc.) [31].

### Western blot

The procedure was performed similarly as described previously [46]. Co-transfected cells were washed with PBS and lysed with RIPA buffer with protease inhibitors (cOm-plete, Roche Diagnostics, Mannheim, Germany). Cell debris was removed by centrifuging at 10,000 g for 10 min. Protein concentration was determined by performing Bradford assay. Then, equal amount of proteins were loaded into 10% polyacrylamide gel and ran at 100 V for 3-4 h. Further, proteins were transferred to PVDF membrane (Millipore, Darmstadt, Germany) at 80 V for 2-3 h. Blocking was done by using 5% non-fat milk. Next, membranes were incubated with primary antibody overnight at 4°C. The blots were washed 3 times with 1× TBST and incubated in their respective secondary antibodies for 1 h at RT. Blots were washed again 3 times with 1 × TBST and developed with Amersham ECL plus (GE Technologies, Buckinghamshire, UK).

### Immunocytochemistry

The immunocytochemistry was performed similarly as described previously [47]. Cells were grown in 12-well plates on coverslips and they were co-transfected with plasmids as described in the result section. Unless stated otherwise, 48 h post-transfection cells were washed with PBS, fixed with 4% paraformadehyde for 30 min at room temperature (RT), and washed 3 times with PBS. Next, cells were permeabilized with 0.1% Triton X for 10 min at RT, blocked with 1% BSA and washed 3 times with PBS. Then, cells were incubated with primary antibody overnight at 4°C and followed by washing with PBS. Next, cells were incubated with respective secondary antibody for 1h at RT and washed 3 times with PBS. Cells were counterstained with DAPI and mounted on a slide. Images were captured using confocal microscope (Zeiss).

### Bioinformatics and statistics

Target genes for miR301 were predicted by the following two computer-aided algorithms: TargetScan (http://www.targetscan.org) and PicTar (http://pictar.mdc-berlin.de). All the statistics (one way and two way ANOVA) were performed using Prism (version 5.0d) and p-value < 0.05 was considered as statistically significant, unless mentioned otherwise. Unless mentioned otherwise, all the presented data represents an average of a minimum of 3 independent experiments.

## SUPPLEMENTARY FIGURES



## Abbreviations

miR: microRNA
PH: pleckstrin-homology
PI3K: phoshatidylinositol-3-kinase
PTEN: phosphatase and tensin homolog
mTOR: mammalian target of rapamycin
DMEM: dulbecco's modified Eagles's medium
RT: room temperature
RIPA: radioimmunoprecipitation assay
PVDF: polyvinylidene fluoride
FBS: fetal bovine serum
7-AAD: 7-aminoactinomycin D
