# Supplementary material for: Inhibition of miR301 enhances Akt-mediated cell proliferation by accumulation of PTEN in nucleus and its effects on cell-cycle regulatory proteins
Source: Oncotarget. 2016 Mar 8;7(15):20953–65. doi: 10.18632/oncotarget.7996 (PMC4991504; doi:10.18632/oncotarget.7996)
Supplement: Supplementary file 1 [file oncotarget-07-20953-s001.pdf]

SUPPLEMENTARY FIGURES

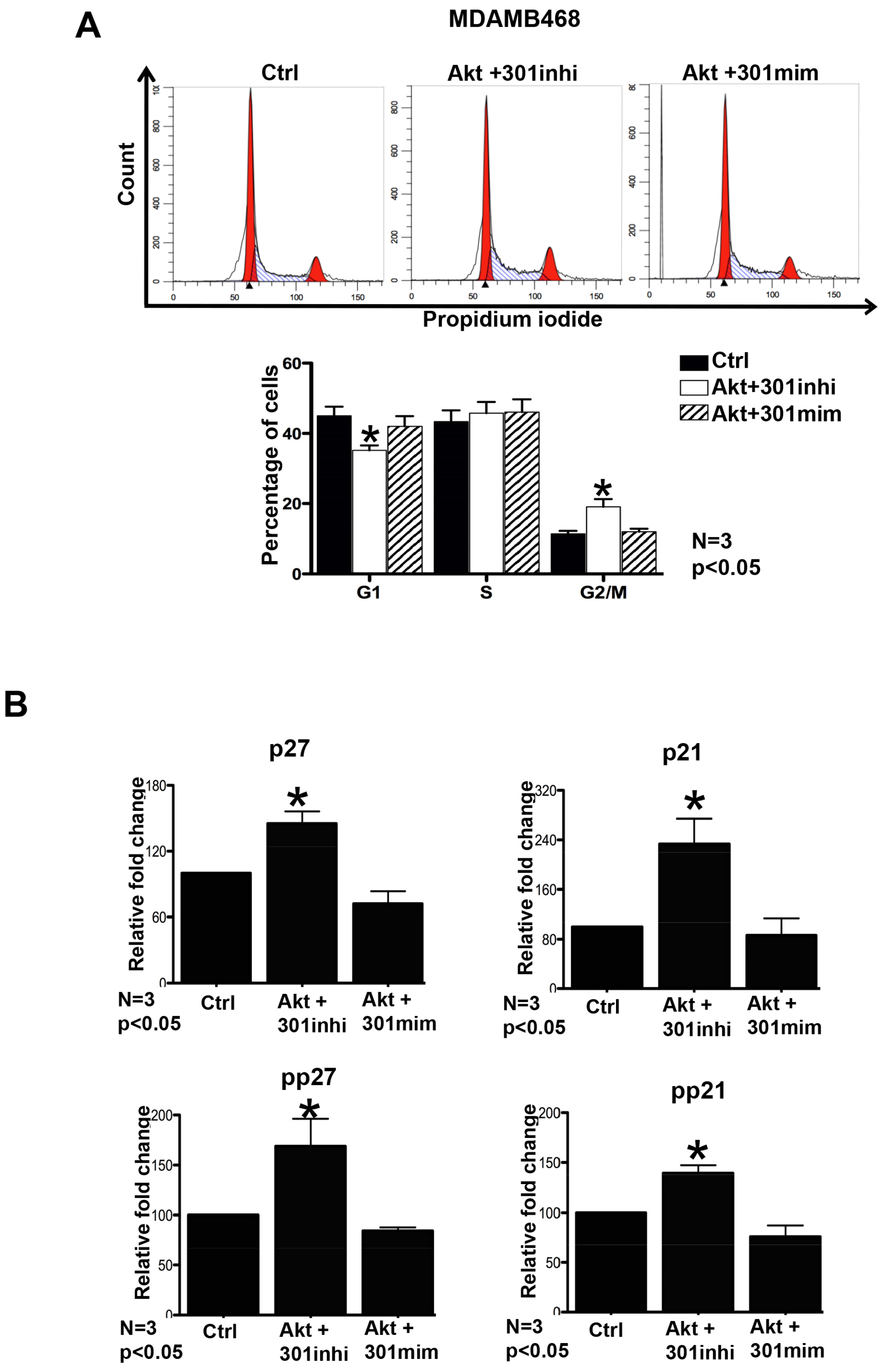

Supplementary Figure S1: Role of miR301 in the presence of Akt on the cell cycle progression. A. Cell cycle analysis shows significant increase in the G2/M phase upon inhibition of miR301 expression in the presence of Akt compared to control and 301mimic+Akt in MDAMB468 breast cancer cells. Lower panel represents quantification of data "A". **B.** Quantitative assessment of $p21^{Waf1/Cip1}$, $pp21^{Waf1/Cip1}$ $p27^{kip1}$ and $pp27^{kip1}$ shows increased protein levels upon miR301 inhibition with Akt compared to control in MCF7 cells (* $p < 0.05$).

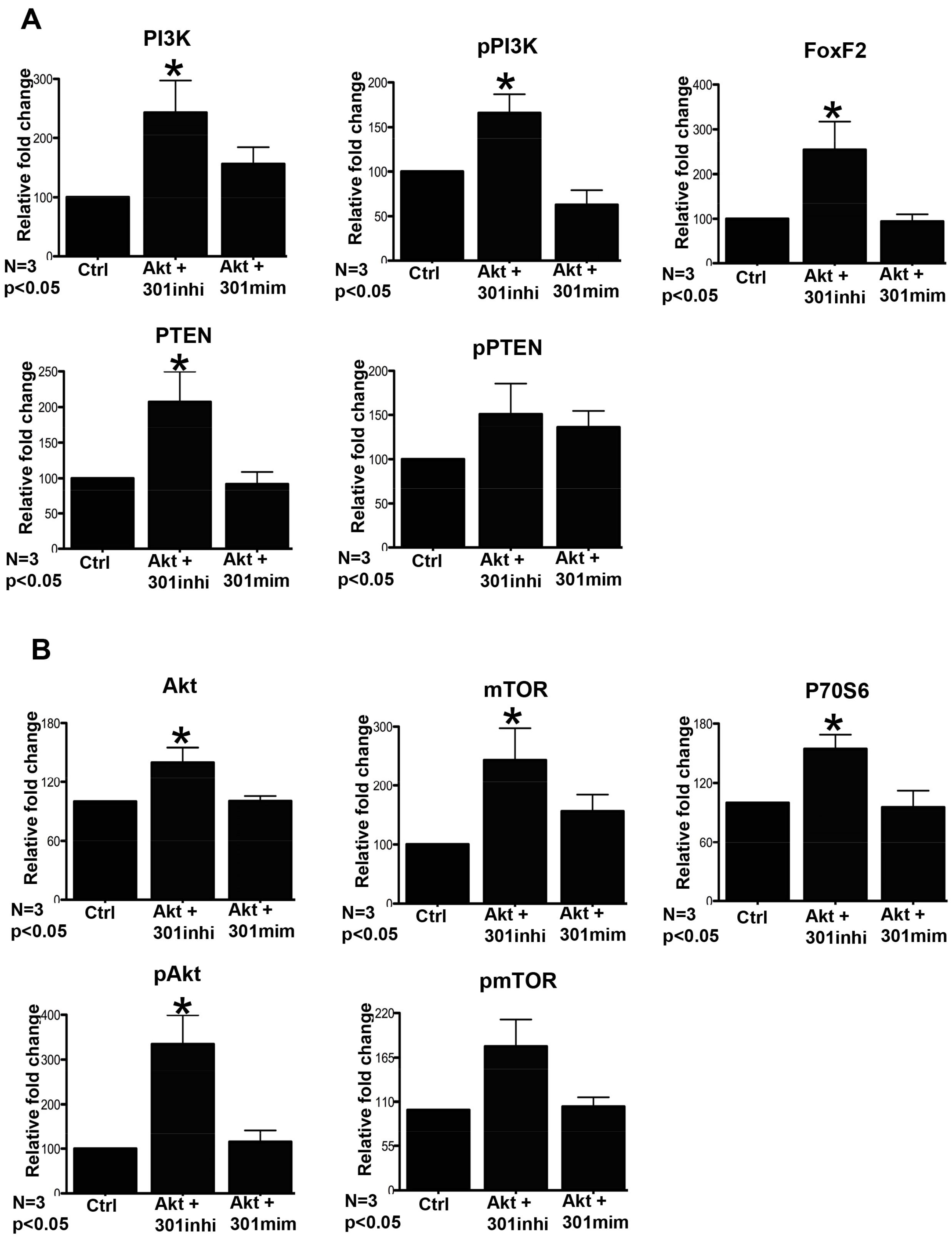

Supplementary Figure S2: Quantification of Western blot results. A. Increased protein expression of PI3K, pPI3K, PTEN and FoxF2 upon miR301 inhibitor with Akt overexpression as compared to control in MCF7 cells. In case of pPTEN, protein expression was not much changed in miR301 inhibitor or mimic with Akt transfected cell in MCF7 cells. **B.** Increased protein expression of Akt, pAkt, mTOR, pmTOR and P70S6 upon miR301 inhibitor with Akt overexpression as compared to control in MCF7 cells (* $p < 0.05$).
